# Insights into the Hypertensive Effects of *Tityus serrulatus* Scorpion Venom: Purification of an Angiotensin-Converting Enzyme-Like Peptidase

**DOI:** 10.3390/toxins8120348

**Published:** 2016-11-24

**Authors:** Daniela Cajado-Carvalho, Alexandre Kazuo Kuniyoshi, Bruno Duzzi, Leo Kei Iwai, Úrsula Castro de Oliveira, Inácio de Loiola Meirelles Junqueira de Azevedo, Roberto Tadashi Kodama, Fernanda Vieira Portaro

**Affiliations:** 1 Immunochemistry Laboratory, Butantan Institute, São Paulo CEP 05503-900, SP, Brazil; daniela.carvalho@butantan.gov.br (D.C.-C.); alexandre.kuniyoshi@butantan.gov.br (A.K.K.); bruno.duzzi@butantan.gov.br (B.D.); pararoberval@gmail.com (R.T.K.); 2 Special Laboratory for Applied Toxinology, Butantan Institute/Center of Toxins, Immune-Response and Cell Signaling (CeTICS), São Paulo CEP 05503-900, SP, Brazil; leo.iwai@butantan.gov.br (L.K.I.); ursula.oliveira@butantan.gov.br (Ú.C.d.O.); inacio.azevedo@butantan.gov.br (I.d.L.M.J.d.A.)

**Keywords:** ACE-like, *Tityus serrulatus* venom, proteases, antivenom, hypertension

## Abstract

The number of cases of envenomation by scorpions has grown significantly in Brazil since 2007, with the most severe cases being caused by the *Tityus serrulatus* scorpion. Although envenomed patients mostly suffer neurotoxic manifestations, other symptoms, such as hypertension, cannot be exclusively attributed to neurotoxins. Omics analyses have detected plentiful amounts of metalloproteases in *T. serrulatus* venom. However, the roles played by these enzymes in envenomation are still unclear. Endeavoring to investigate the functions of scorpion venom proteases, we describe here for the first time an Angiotensin I-Converting Enzyme-like peptidase (ACE-like) purified from *T. serrulatus* venom. The crude venom cleaved natural and fluorescent substrates and these activities were inhibited by captopril. Regarding the serum neutralization, the scorpion antivenom was more effective at blocking the ACE-like activity than arachnid antivenom, although neither completely inhibited the venom cleavage action, even at higher doses. ACE-like was purified from the venom after three chromatographic steps and its identity was confirmed by mass spectrometric and transcriptomic analyses. Bioinformatics analysis showed homology between the ACE-like transcript sequences from *Tityus* spp. and human testis ACE. These findings advance our understanding of *T. serrulatus* venom components and may improve treatment of envenomation victims, as ACE-like may contribute to envenomation symptoms, especially the resulting hypertension.

## 1. Introduction

According to Brazil’s Ministry of Health, since 2007 scorpion stings have been the main form of envenomation by animals in this country. An epidemiological survey conducted by the Ministry of Health shows that, between 2010 and 2013, cases of scorpion envenomation represent 49% of poisonings by venomous animals in Brazil, surpassing those by snakes (17%) and spiders (18.5%). This scenario is mainly attributed to the proliferation of *Tityus serrulatus* scorpions, synanthropic animals that reproduce by parthenogenesis [1] and whose potent venom contributes to the occurrence of critical clinical envenomation. Thus, *T. serrulatus* venom (*Ts*v) is one of the most studied Brazilian scorpion venoms [2].

Neurotoxins in *Ts*v cause most of the envenomation symptoms by promoting neurotransmitter release from the autonomic nervous system and the adrenal medulla onto several organs [3]. These events result in clinical manifestations such as restlessness, excessive salivation, lacrimation, hypertension followed by hypotension, heart failure, and cardiogenic shock, among others. Not all symptoms, however, are attributed to a direct action of specific neurotoxins on organs [4], and recent studies suggest that other molecules may contribute to these effects [5,6,7]. Metallopeptidases, hyaluronidase, biogenic amines, antimicrobial peptides (AMP), and other oligopeptides are also present in *Ts*v [7,8,9]; however, the role of these molecules in envenomation is still unclear.

Peptidases present in animal venoms often play an important role in the envenomation syndrome, as they can cleave proteins and peptides that are key factors for physiological systems. For instance, in snake venoms, serine peptidases can affect the coagulation cascade and kallikrein–kinin system, while metallopeptidases target components of blood coagulation and platelet aggregation, both resulting in an imbalance of the hemostatic system [10,11]. Although snake venom peptidases are the most studied, due to their abundance, proteolytic activity has recently been described in jellyfish [12], cone snail [13], wasp [14], spider [15], and scorpion [16] venoms.

Thus far, metallopeptidases and hyaluronidases comprise the only classes of enzymes purified and with their functional activities determined in *Tityus* spp. venoms [6,9], although transcriptomic studies have identified other classes of enzymes as well [8,17]. Studies from our group have shown that *Ts*v proteases cleave mammal peptides in vitro, and that this activity is inhibited by high concentrations of commercially available antivenoms used in the treatment of envenomed patients in Brazil [16,18]. Moreover, endopeptidases, aminopeptidases, and carboxypeptidases that cleave endogenous peptides from the *Ts*v and inactivate human bioactive peptides have been detected [18,19]. One of these, antarease, is a metallopeptidase ubiquitous in the venom of scorpions of family Buthidae [20], to whose action the pancreatitis observed in mice after venom injection is attributed [21].

The human somatic angiotensin-I converting enzyme (sACE, EC 3.4.15.1) is a dipeptidyl carboxypeptidase that plays a crucial role in blood pressure regulation through the renin–angiotensin (RAS) and kalikrein–kinin (KKS) systems. sACE converts angiotensin I into angiotensin II in RAS, and inactivates bradykinin in KKS, leading to blood pressure increase. sACE has two highly similar, independent catalytic domains, the *N*- and *C*-terminal domains. Although both have proteolytic activity, regulation of blood pressure mostly depends on the *C*-terminal domain, as it is responsible for producing angiotensin II [22]. A smaller isoform of sACE is also present in mammals: known as testicular ACE (tACE), it contains only the *C*-terminal domain [23,24]. The same gene encodes both somatic and testicular ACE [23,25] but tACE is only found after puberty in male germinal cells, where it participates in sperm maturation, in contrast with the constant and wide expression of sACE that acts all over the body [26].

ACE-like enzymes are highly conserved, having also been detected in insects, where they play a role in feeding, reproduction, and the production of peptide hormones and neurotransmitters [27]. Also, functional ACE-like activity was detected in animal venoms of the fish-hunting cone snail [13], vampire snail [28], and solitary endoparasitic wasp [14]. In scorpions, ACE-like peptidases were detected by transcriptome analysis in *Hottentotta judaicus* [29], *Tityus stigmurus* [30], and *Tityus bahiensis* [17] venoms.

Here we describe and characterize for the first time an Angiotensin I-Converting Enzyme-like peptidase activity in *Tityus serrulatus* venom and the evaluation of commercially available antivenoms to neutralize it. We used proteolytic activity assays to detect an ACE-like peptidase, then purified and confirmed its identity by tryptic digestion/mass spectrometric and transcriptomic analysis.

This report may contribute to our understanding of the role of proteases from scorpion venoms in the envenomation process, as an ACE-like peptidase may contribute to the hypertension observed in human victims.

## 2. Results

### 2.1. FRET Substrates Specific for Carboxy- and Endopeptidases on Ts*v*

**Figure 1:**
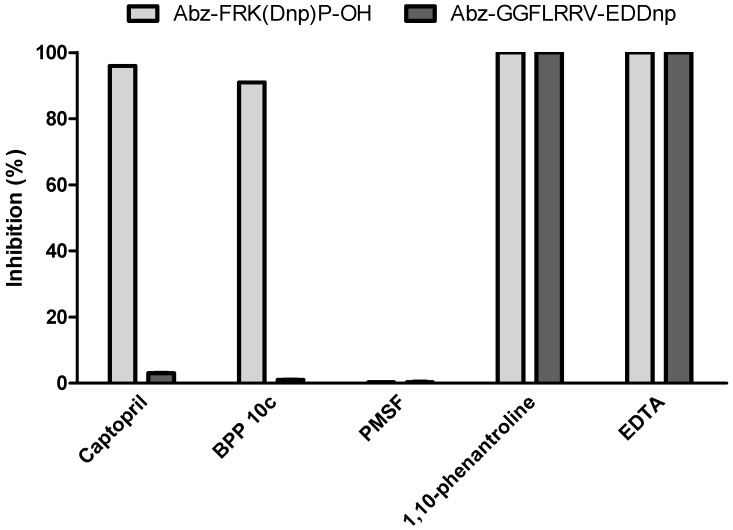
Fluorimetric assays for carboxy- and endopeptidases activities on *Ts*v using peptidase inhibitors. Captopril (100 nM), BPP 10c (16 µM), EDTA (50 mM), 1,10-phenantroline (2 mM), PMSF (2 mM) were tested on the whole *Tityus serrulatus* venom (1 µg) in a fluorometric assay with Abz-FRK(Dnp)P-OH (dark grey) and Abz-GGFLRRV-EDDnp (light grey). The reactions occurred in 100 mM Tris, 50 mM NaCl, 10 µM ZnCl_2_ buffer, pH 7.0 at 37 °C. Experiments were done in duplicate. The SD of kinetic results in each case was never greater than 5% of the value obtained.

We first evaluated *Ts*v activity with peptidase inhibitors, using two fluorescent substrates: Abz-FRK(Dnp)P-OH, which was designed for ACE studies [31], and Abz-GGFLRRV-EDDnp, homologous to the dynorphin 1–13 sequence and previously determined as substrate for *Ts*v endopeptidases [18]. Results are presented in Figure 1.

Although only metallopeptidases were detected in *Ts*v (due to inhibition of EDTA and 1,10-phenantroline), we observed that different proteases act on each substrate. For Abz-FRK(Dnp)P-OH, the two classical ACE inhibitors, captopril (100 nM) and BPP 10c (16 µM), significantly inhibited *Ts*v activity (98% and 90%, respectively), while Abz-GGFLRRV-EDDnp hydrolysis was not affected by these inhibitors at the same concentrations. However, PMSF (2 mM) had no effect on the activity of the venom on any fluorescent substrates tested. Thus, it seems that Abz-GGFLRRV-EDDnp is cleaved mainly by metalloendopeptidases and Abz-FRK(Dnp)P-OH by metallo dipeptidyl carboxypeptidases.

### 2.2. In Vitro Serum Neutralization Assays Using Abz-FRK(Dnp)P-OH as Substrate

**Figure 2:**
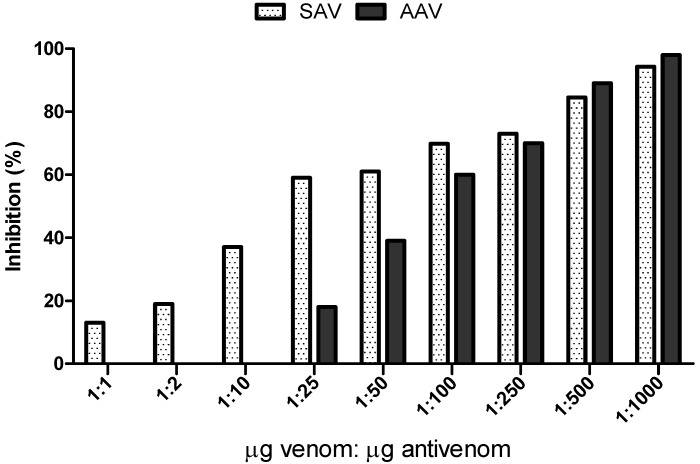
In vitro serum neutralization of Abz-FRK(Dnp)P-OH hydrolysis by *Ts*v using scorpion (SAV) and arachnid (AAV) commercial antivenoms. The venom was incubated for 30 min at room temperature with nine concentrations of antivenom (weight ratio of venom:antivenom)—1:1; 1:2; 1:10; 1:25; 1:50; 1:100; 1:250; 1:500, and 1:1000—and then the fluorescent substrate was added. The result is expressed as % inhibition of peptidase activity in the venom (%). The result represents the mean of two independent experiments. The SD of kinetic results in each case was never greater than 5% of the value obtained.

We tested nine different concentrations of arachnid (AAV) and scorpion (SAV) antivenoms to determine their efficacy in neutralizing *Ts*v hydrolysis of Abz-FRK(Dnp)P-OH (Figure 2).

SAV partially inhibited the activity even at low venom:antivenom ratios, whereas AAV started inhibiting *Ts*v at a higher dose, after 25 µg of antivenom (1:25), but also reached higher levels of inhibition than SAV at concentrations of 1:500 µg. Neither antivenom fully neutralized *Ts*v activity at the highest dose tested (1:1000), with inhibition reaching 94% for SAV and 98% for AAV.

### 2.3. Effect of Chloride Ion Concentration on Ts*v* and sACE

**Figure 3:**
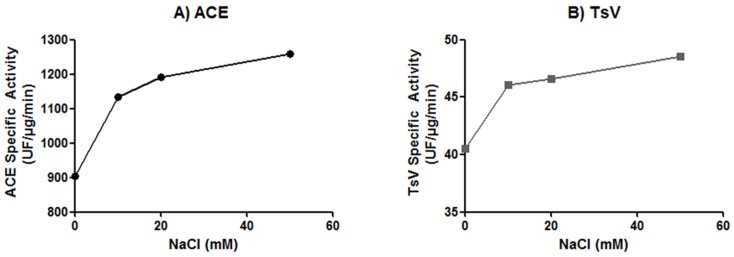
Effect of chloride ions concentration on (**A**) angiotensin-converting enzyme (ACE) and (**B**) *Tityus serrulatus* venom. For both, Abz-FRK(Dnp)P-OH hydrolysis was determined in Tris 100 mM, ZnCl_2_ 10 µM buffer, with four different concentrations of NaCl: 0, 10 mM, 20 mM, and 50 mM. The result represents the mean of two independent experiments. The SD of kinetic results in each case was never greater than 5% of the value obtained.

As chloride ions are known to affect ACE activity [32], we compared *Ts*v and ACE hydrolysis of the Abz-FRK(Dnp)P-OH substrate in the presence of different Cl^−^ concentrations (Figure 3).

As showed in Figure 3, both enzymes were already active in the absence of NaCl, with higher proteolytic activities observed as NaCl concentrations increased. At 10 mM NaCl, *Ts*v and ACE activities increased by 15% and 25%, respectively, and, at the highest NaCl concentration tested (50 mM) hydrolysis of Abz-FRK(Dnp)P-OH by *Ts*v increased by more than 35%, and around 46% by ACE.

### 2.4. Hydrolysis of ACE Natural Substrates by Ts*v*

Angiotensin I and bradykinin were incubated with *Ts*v in the classical ACE working buffer, 100 mM Tris, 50 mM NaCl, 10 µM ZnCl_2_, pH 7.0. After verifying, with reserve phase chromatography, that both peptides were substrates for *Ts*v, we manually collected the products of hydrolysis and analyzed them by mass spectrometry. Additionally, we determined the specific activity of the venom on each peptide, including hemopressin, and determined the cleavage sites (Table 1).

Hemopressin was the best substrate for the whole venom (0.40 µM/µg/min), followed by angiotensin I (0.05 µM/µg/min) and bradykinin (0.045 µM/µg/min). One of the fragments collected from angiotensin I corresponded to angiotensin II, due to the removal of His^9^-Leu^10^. Other fragments were also formed from angiotensin I hydrolysis, such as Ang_(1–4)_, Ang_(5–10)_, Ang_(1–7)_, and Ang_(8–10)_, probably due to endopeptidase activity; and Ang III and Ang_(6–10)_, probably due to aminopeptidase activity. Bradykinin hydrolysis by the venom formed the fragments BK_(1–7)_, BK_(1–5)_, the expected products of ACE-like activity, and BK_(3–7)_. Hemopressin cleavage sites were determined previously [18], but the hydrolysis rate increased 5.6 times when the buffer containing 10 µM ZnCl_2_ was used.

### 2.5. Inhibition Assay on Reverse Phase Chromatography

As new substrates and hydrolysis rates were observed for *Ts*v when using the ACE buffer, we evaluated inhibition by captopril, a potent and specific human ACE inhibitor, and by EDTA (Table 2).

**Figure 4:**
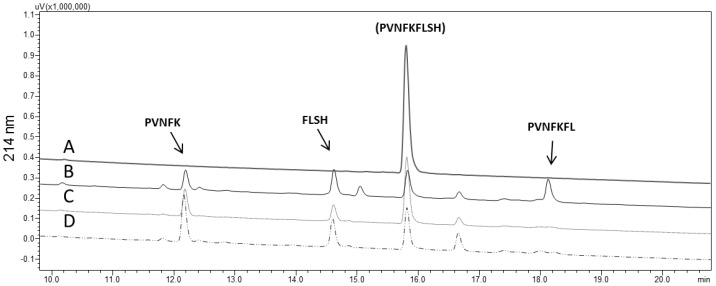
Hemopressin hydrolysis by *Tityus serrulatus* venom on RP-HPLC. (**A**) Hemopressin, 30 µM, without venom; (**B**) hemopressin after 2 h incubation with *Ts*v (1 µg), and its fragments; (**C**,**D**) hemopressin hydrolyzed by *Ts*v in presence of 100 nM and 1 µM of captopril, respectively, showing that the product (PVNFKFL) is not formed. Experiments were done in duplicate.

EDTA fully inhibited the activity of *Ts*v on all substrates. Captopril, in either of the two concentrations tested, failed to inhibit the cleavage of dynorphin 1–13 in any concentration, which was expected, as a similar result was observed with the analog fluorescent substrate, Abz-GGFLRRV-EDDnp (Figure 1). On the other hand, captopril inhibited bradykinin and angiotensin I hydrolyses in a dose-dependent manner (Table 2). Hydrolysis of hemopressin was also strongly inhibited (37.8%) by 100 nM captopril, but less so with a higher dose (1 µM) of this inhibitor. The RP-HPLC profile of hemopressin hydrolysis by *Ts*v (Figure 4), shows that, when captopril was present (in both doses), one of the products (PVNFKFL) was not formed, indicating that the dipeptidyl carboxypeptidase activity of the whole venom was abolished. Nevertheless, the other products (PVNFK and FLSH) were not affected by this inhibitor.

### 2.6. Purification of ACE-Like Peptidase from *T. serrulatus* Venom

**Figure 5:**
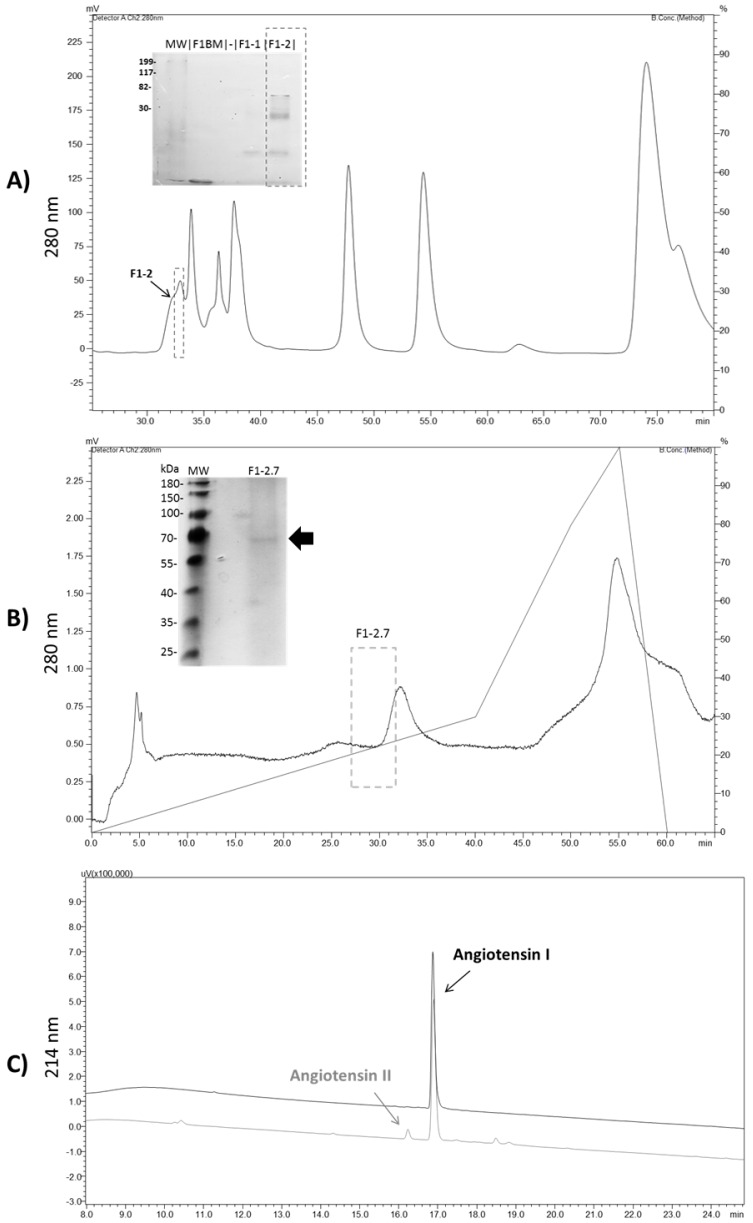
Purification of ACE-like enzyme from *Tityus serrulatus* venom. (**A**) Fraction 1 was fragmented in gel filtration Diol-300 column, and F1-2 was the only fraction able to cleave the FRET substrate. SDS-PAGE showed a separation of low molecular weight bands. (**B**) Profile of the fraction F1-2 on a cation exchange column (black line), with F1-2.7 being the fraction with the highest peptidase activity, and resulting in a single protein band in 13% SDS-PAGE. In order to maintain the activity, after each step the buffer was immediately changed to 100 mM Tris, 50 mM NaCl, 10 µM ZnCl_2_ buffer, pH 7.0, using a 10 kDa molecular weight cutoff membrane. The grey line represents the NaCl gradient. (**C**) Conversion of angiotensin I into angiotensin II by the purified ACE-like enzyme. The details of the experiments are described in Section 4.8.

In order to purify the ACE-like peptidase present in *Ts*v, we performed three chromatographic steps to obtain a homogeneous and active enzyme. First, the whole *Ts*v was applied to an HPLC-DEAE column, and the ACE-like activity was identified (77 UF/µg/min) in fraction 1, which did not interact with the column (data not show). Then, fraction 1 was loaded onto a Diol-300 column, from which fractions were collected (Figure 5, panel A) and screened by Abz-FRK(Dnp)P-OH hydrolysis. Since fraction F1-2 showed the best hydrolysis rates (88 UF/µg/min), it was further fractioned with PA-CM ion exchange chromatography. After this last step, we observed one active fraction, presenting a single band of 70 kDa (Figure 5, panel B), with a specific activity of 220 UF/µg/min on the FRET substrate. In the final chromatography step, the purification factor obtained was 22.9 with a yield of 1% (Table S1).

**Figure 6:**
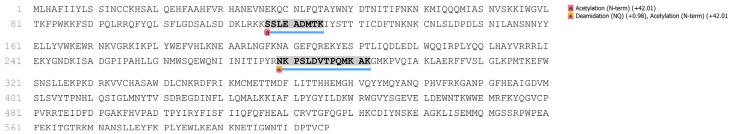
Tryptic peptides from fraction F1-2.7 that matched with the predicted ACE-like from *T. serrulatus* (GenBank TserSP00939) obtained from transcriptomic analysis. The peptides were found using Peaks DB with FDR ≤ 1%.

The protein content from the SDS-PAGE band was extracted and subjected to MS/MS for peptide fingerprint analysis. Using a database that combined sequences restricted to *Tityus* genus from UNIPROT and the transcript sequences of ACE-like peptidase from *Tityus* scorpion venom glands, PeaksDB was able to identify, with high confidence (FDR ≤ 1%), two unique ACE-like peptides from *Tityus serrulatus* (TserSP00939) (Figure 6). Additionally, the pure enzyme was able to convert angiotensin I into angiotensin II, in addition to Abz-FRK(Dnp)P-OH, with specific activity of 0.01 µM/µg/min (Figure 5, panel C).

### 2.7. Sequence Analysis

We aligned the amino acid sequences of ACE-like peptidases obtained from transcriptomics data analysis from *Tityus serrulatus, T. obscurus* and *T. bahiensis* with each other and with human testicular ACE (Table 3).

The identity with human tACE varied from 23% to 39%; however, the metallopeptidase motif was highly conserved throughout the species (Figure S1). The ACE-like sequences of *Tityus serrulatus* (TserSP00939) and *Tityus obscurus* (Tobs01141) had 92.51% of similarity, while the *tbah*ACE-like sequence shared 62.31% and 67.34% similarity with *T. obscurus* and *T. serrulatus*, respectively.

## 3. Discussion

Using enzymatic assays, protein purification, mass spectrometry analysis, and comparative studies of the transcript sequences in the venom glands of *Tityus* spp*.* scorpions, we show for the first time the presence of an ACE-like peptidase in the venom of *Tityus serrulatus*.

The venom cleaves physiologically important ACE substrates, such as angiotensin I, bradykinin, and hemopressin, and captopril inhibits this activity. Expected products from these hydrolyses were observed, such as hemopressin 1–7, angiotensin II, BK_1–5_, and BK_1–7_, consistent with the ACE specificity for these substrates [33,34]. However, the presence of other fragments indicates the participation of more venom proteases in the cleavage of these substrates. In addition, Abz-FRK(Dnp)P-OH, a substrate specifically designed for ACE [31], was an important tool to identify and purify the ACE-like peptidase on *T. serrulatus* venom, along with the successful ACE inhibition by captopril and BPP 10c [31,35].

At the end of the third chromatography step, we observed a single protein band in the silver-stained gel with molecular mass around 70 kDa that converts angiotensin I into angiotensin II. Thus, the ACE-like enzyme present in *Ts*v has a molecular mass similar to tACE. Besides the precursor of ACE-like peptidase being sequenced by RNA sequencing (GenBank TserSP00939) and the identification of the ACE-like peptidase from *T. serrulatus* through mass spectrometric analysis, the isoform sequences are also present in transcripts from the venom gland of *T. bahiensis* (GenBank JAG85170.1), *T. stigmurus* [30], and *T. obscurus* (GenBank Tobs01141 and GenBank Tobs00978) [36]. Thus, these sequences helped to validate the purification of this enzyme, achieving two unique peptides from *T. serrulatus*’s ACE-like sequence. Moreover, the alignment indicated high homology between the ACE sequences from Brazilian *Tityus* species and a conserved metallopeptidase motif HHEMGHV with human tACE.

The expressive number of ACE-like enzymes described in invertebrates indicates that this metallopeptidase is conserved throughout evolution [27]. Also, in accordance with our results, the ACE-like enzymes in invertebrates include only a C-domain, similar to the human testicular form of the enzyme. Despite the many studies with ACE in invertebrates, natural substrates for these enzymes are yet to be described [27], and, therefore, their physiological role still needs clarification. In general, ACE-like peptidases show broad substrate specificity, cleaving a large number of peptides in vitro [33]. The exceptions are peptides with proline residues at the penultimate position, such as BPPs [37].

Studies have shown that male fruit flies with mutations in the ACE gene are sterile [38]. Other studies suggest a possible physiological role of ACE-like peptidases in the midgut of invertebrates, but solely based on their presence in this region [39,40]. Also, the presence of ACE-like enzymes in the neuropil area of insect brains, and the ability of the enzyme to cleave insect tachykinin (Lom TK-1) in vitro, may indicate that this enzyme participates in the processing of neuropeptides [41,42]. In this scenario, we hypothesize that the ACE-like peptidase present in *T. serrulatus* venom contributes to the ability to capture or kill prey.

Moreover, the presence of this enzyme in the venom may be related to forming endogenous peptides in *Ts*v, since a proteomic study identified post-translational modifications of toxins in this venom and showed that about 80% of the venom molecules are degraded by endogenous peptidases, particularly amino and carboxypeptidases [19]. Also, a recent report from our group showed that metallocarboxypeptidases contribute to inactivate a series of human bioactive peptides [18].

Lastly, we cannot exclude the involvement of this carboxypeptidase in the envenomation. According to Safavi-Hemami and collaborators (2013), who described an angiotensin I-converting enzyme in the venom of cone snails, this enzyme may promote vasoconstriction of blood vessels at the sting site by angiotensin II, along with catecholamine [13]. In fact, animals and patients envenomed by scorpions from the Buthidae family present elevation of plasma renin–angiotensin activity and, consequently, an increase of angiotensin II [43,44]. This observation might, at least in part, be explained by the action of the venom ACE-like enzyme, as we showed here that it is capable of releasing angiotensin II, and victims of *Tityus serrulatus* sting present transient hypertension [45,46]. Until now, neurotoxins were considered the main cause of hypertension in stung patients, due to catecholamine release [7]. However, as Ang II can also stimulate the release of catecholamines, this may contribute synergistically to the hypertension observed in human victims [47,48].

Besides Ang II formation by the whole venom and the purified peptidase, we also observed that *Ts*v acts on Ang I, releasing Ang III, a hypertensive peptide with activity comparable to Ang II [49]. Differently, Ang 1–7, another product of the action of *Ts*v, promotes vasodilation, which would seem to be a contradictory action of the venom. However, envenomed patients commonly present persistent hypotension after the transient hypertension [7]. Heretofore, hypotension could be explained by the presence of hypotensins, which are bradykinin-potentiating peptides that do not inhibit human ACE activity [50]. Our results suggest yet another factor that can contribute to this effect. In addition, and since animal venoms aim to destabilize homeostasis in the prey, the joint action of multiple molecules in the venom (neurotoxins, proteases, among others) is important to cause the envenomation syndrome.

Furthermore, taking into account the possible involvement of ACE-like during the envenomation, especially on hypertension, the in vitro serum neutralization of *Ts*v is an important assay. The antivenom sera represent the only specific therapy available and recommended by the World Health Organization (WHO) to treat incidents involving animal venoms. In case of scorpion envenomation, the administration of either scorpion (SAV) or arachnid (AAV) antivenoms is prescribed; in the most severe cases, it is recommended to use 4–6 ampoules. Our results show that SAV and AAV produced different levels of neutralization: while SAV caused partial inhibition at lower doses, AAV was slightly more efficient at the highest doses. The effect of the lower SAV concentration is probably due to ACE-like peptidase being present in both scorpion venoms used for the SAV immunization pool (*T. serrulatus*, 50% and *T. bahiensis*, 50%), as is shown in Figure S1, while the venoms used to obtain arachnid antivenom include only one scorpion species (*T. serrulatus*, 57%), together with two spider species (*Phoneutria nigriventer* and *Loxosceles gaucho*). The inhibition observed using high doses of both antivenoms could be a result of some sort of interaction impairment between the ACE-like and the FRET substrate due to the high number of non-specific antibodies, and that might not represent a real neutralization by the antivenom. However, the use of higher doses of antivenom may be injurious to patients, as it can cause adverse reactions known as “serum sickness” [51].

In summary, here we describe, for the first time, the purification and characterization of ACE-like enzyme in *Tityus serrulatus* venom, as well as two fluorescent substrates specific for endo- and carboxypeptidases in this venom. Also, since we observed formation of Ang II in vitro, we hypothesize that this enzyme may also contribute to the hypertension observed in the envenomation syndrome, along with other molecules. Considering that scorpion bites are a critical public health problem in Brazil, this study demonstrates the importance of characterizing new molecules related to envenomation processes as well as evaluating antivenom therapies. The high concentrations of commercially available antivenoms necessary to partially block the ACE-like activity in *Ts*v may be harmful to victims.

## 4. Materials and Methods

### 4.1. Reagents

Phenylmethanesulfonylfluoride (PMSF), 1,10-phenanthroline, dynorphin 1–13 (Dyn 1–13), hemopressin (hemo), angiotensin I (Ang I), bradykinin (BK), captopril, and rabbit lung somatic angiotensin I-converting enzyme (ACE) were purchased from Sigma-Aldrich (St. Louis, MO, USA). Acetonitrile and trifluoroacetic acid (TFA) were acquired from J.T. Baker. The fluorescent resonance energy transfer (FRET) substrates Abz-GGFLRRV-EDDnp and Abz-FRK(Dnp)P-OH, synthesized with automated solid-phase synthesis [52], were kindly provided by Dr. Adriana Carmona, from the Department of Biophysics of UNIFESP-EPM, São Paulo, Brazil.

### 4.2. Venoms and Antivenoms

The lyophilized venom of *Tityus serrulatus* (Batch No. 2146 and batch No. 744) were provided by the Venom Section of Instituto Butantan, SP, Brazil. The venoms were submitted to a 10 kDa molecular weight cutoff (Amicon Ultra-15 Centrifugal Filter Devices) and stock solution was prepared in 50 mM sodium phosphate and 50 mM NaCl, pH 6.0. The scorpion and arachnid antivenoms (SAV and AAV, respectively) were from the Hyperimmune Plasmas Processing Section, Instituto Butantan, SP, Brazil. The SAV (batch No. 0905104/A) and the AAV (batch No. 0706121) protein concentrations were 8.43 g/dL and 15.4 g/dL, respectively. The antivenoms from Instituto Butantan are produced through hyperimmunization of horses, with a pool of *T. serrulatus* (50%) and *T. bahienses* (50%) venoms for SAV, and *T. serrulatus* (57%), *Phoneutria nigriventer* (21.5%) and *Loxosceles gaucho* (21.5%) venoms for AAV.

### 4.3. Fluorescent Substrate-Specific for Carboxy- and Endopeptidases, and Peptidase Inhibitors

The substrate-specific assay was carried out using 1 µg *Tityus serrulatus* venom (*Ts*v) and the fluorescent substrates Abz-GGFLRRV-EDDnp (5 µM) [18] and Abz-FRK(Dnp)P-OH (4 µM), in 100 mM Tris, 50 mM NaCl, 10 µM ZnCl_2_ buffer, pH 7.0. All reactions were monitored by measuring hydrolyses using a fluorimeter (Victor 3™, Perkin-Elmer, Waltham, MA, USA; λem 420 nm and λex 320 nm), at a stable temperature of 37 °C. The measurements of peptidase activity were made for 15 min continuously (one read per minute). The fluorometric assays were analyzed using Grafit 5.0 from Erithacus Software (version 5.0.6, 1989-2003, Erithacus Software, West Sussex, UK), and the hydrolysis rates (UF/min) were determined. All fluorometric measurements were made in duplicate, and the results are shown as the mean with SD.

For the inhibition of *Ts*v, we used the serine peptidase inhibitor PMSF (2 mM), the metallopeptidase inhibitors 1,10-phenantroline (2 mM) and EDTA (50 mM), and the peptidase-specific inhibitors captopril (100 nM) and BPP 10c (Bradykinin Potentiating Peptide, <ENWPHPQIPP, 16 µM). Control samples included a volume of ethanol equal to that used in the PMSF and 1,10-phenantroline stock solutions. PMSF and 1,10-phenantroline were pre-incubated for 30 min at room temperature before the test.

### 4.4. Effect of Chloride Ion Concentration on Ts*v* and ACE Activities

The effects of ion chloride on *Ts*v were evaluated in parallel with angiotensin I-converting enzyme (ACE) on fluorometric experiments. For this, *Ts*v and ACE were added to 96-well plates at concentrations of 1 µg and 50 ng, respectively, in 100 mM Tris, 10 µM ZnCl_2_, pH 7.0 buffer, at a final volume of 100 µL, with 4 µM Abz-FRK(Dnp)P-OH. Four concentrations of NaCl were tested on *Ts*v and ACE: 0 (control), 10 mM, 20 mM, and 50 mM. All the assays were performed in triplicate, and the specific venom peptidase activities were expressed as units of free fluorescence of the cleaved substrate per µg of venom per min (UF/µg/min).

### 4.5. In Vitro Serum Neutralization Assays Using Abz-FRK(Dnp)P-OH

The serum neutralization assay of *Ts*v activity upon the Abz-FRK(Dnp)P-OH substrate was performed using different doses of the antivenoms. The *Ts*v (1 µg) was incubated at room temperature with the antivenoms for 30 min in the following concentrations (weight ratio of venom and antivenom, respectively): 0 (control), 1:1; 1:2; 1:10; 1:25; 1:50; 1:100; 1:250; 1:500, and 1:1000. After incubation, the FRET substrate was added, and the residual peptidase activity of the venom measured, in duplicate, as described (Section 4.3).

### 4.6. Cleavage of Biologically Active Peptides and Inhibition by Captopril

*Ts*v (0.5 µg) was incubated in 100 mM Tris, 50 mM NaCl, 10 µM ZnCl_2_ buffer, pH 7.0, at 37 °C with dynorphin 1–13 (30 µM), for 70 min; hemopressin (30 µM) for 2 h; angiotensin I (30 µM) for 4 h; and bradykinin (30 µM) for 6 h. Captopril was used at 100 nM and 1 µM final concentrations. EDTA was also used at 100 mM final concentration. All experiments were performed in duplicate, and the results are shown as the mean with SD. Hydrolyses were analyzed by reverse-phase HPLC (Prominence, Shimadzu, Japan), with 0.1% trifluoroacetic acid (TFA) in water, as solvent A, and acetonitrile and solvent A (9:1), as solvent B. Separations were performed at a flow rate of 1 mL/min, using a Restek Ultra C-18 column (4.6 mm × 250 mm) and a 20%–60% gradient of solvent B over 30 min. In all cases, elution was followed by measurement of ultraviolet absorption (214 nm). The specific activities were expressed in µM of hydrolyzed substrate per µg of venom per minute (µM/µg/min) and the inhibition of venom peptidase activity was calculated by comparing the peptide areas.

### 4.7. Mass Spectrometry Analysis of Ang–I and BK Fragments Produced by Ts*v*

The products of the hydrolysis of angiotensin-I and bradykinin, catalyzed by *Ts*v, were manually collected and subjected to mass spectrometry analysis. The reverse phase chromatography fractions were resuspended in 0.1% formic acid and analyzed by online liquid chromatography in an Easy-nLC Proxeon nanoHPLC system coupled to an LTQ-Orbitrap Velos (Thermo Fisher Scientific, Bremen, Germany) through a nanoelectrospray ion source. Separation was carried out in a 10-cm column (75 µm i.d. × 350 µm e.d.) packed in-house with 5-µm Jupiter^®^ C-18 beads (Phenomenex, Torrance, CA, USA). Peptides were eluted with a linear gradient of 5%–30% acetonitrile, in 0.1% formic acid, in 45 min at 300 nL/min. The spectrometer was operated in data-dependent mode and the 10 most intense peaks were selected for CID fragmentation after acquiring each full scan. The settings for the spectrometer were defined as: high-resolution full MS parameters (1 μscan; full MS mass range *m*/*z* of 200–2000 with an *R* = 30,000 and a target value of 1× 104 ions; max injection time = 10 ms). For fragment scans the settings were: an isolation window of 2 Da, a max list size of 500, a time window of 30 s, a minimum signal of 5000, activation time = 10 ms and normalized collision energy = 35%.

### 4.8. Data Analysis

The raw data files were submitted to searches against the angiotensin I and bradykinin sequences using PEAKS Studio (version 8, Bioinformatics Solution, Waterloo, ON, Canada) [53,54]. A decoy database was also searched to calculate False Discovery Rate (FDR) using the decoy-fusion method [53,54]. The search parameters were: no enzyme specificity; precursor mass tolerance set to ±10 ppm and a fragment ion mass tolerance of ±0.5 Da; oxidized methionine (M + 15.994915 Da) was set as variable modification. The identified peptides were then sorted by their Average of Local Confidence (ALC) to select the best spectra to annotate, and were filtered by FDR ≤ 1%.

### 4.9. Purification of ACE-Like Peptidase from *Tityus serrulatus* Venom

All purification steps were followed by buffer exchange to 100 mM Tris, 50 mM NaCl, 10 µM ZnCl_2_ pH 7.0, using a 10 kDa molecular weight cut off (Amicon Ultra-15 Centrifugal Filter Devices), taking into account that ACE activity is sensitive to chloride concentrations [32]. Moreover, the protein content of all samples was quantified with the commercial kit Quick Start™ Bradford Protein Assay (Bio Rad, Hercules, CA, USA). In order to screen for ACE-like activity, each collected fraction was tested with a fluorometric assay (5% of final volume) using the fluorescent substrate Abz-FRK(Dnp)P-OH (4 µM), as described in Section 4.3.

The lyophilized *Tityus serrulatus* venom was dissolved in 5 mL of 20 mM Tris, 20 mM NaCl, pH 8.2 buffer (final concentration 10 mg/mL). *Ts*v was first submitted to anion exchange chromatography in an HPLC system (Shimadzu Co., Kyoto, Japan) using a Shim-Pack PA-DEAE column (20 mm × 100 mm) at 5 mL/min flow. The gradient used was 0%–40% B for 40 min (buffer A containing 20 mM Tris, 20 mM NaCl, pH 8.2 and buffer B composed of buffer A containing 500 mM NaCl, pH 8.2). Next, fraction 1 was applied to a Shim-pack Diol-300 (7.9 mm × 50 cm) gel filtration column coupled to an HPLC system (Shimadzu Co., Kyoto, Japan) and was eluted with 200 mM sodium sulfate, 10 mM sodium phosphate, pH 7.0 buffer at 0.5 mL/min flow rate. Then, F1-2, which is the active fraction upon the FRET substrate, was injected into a cation exchange Shim-Pack PA-CM column (20 mm × 100 mm) in an HPLC system (Shimadzu Co., Kyoto, Japan) at a flow rate of 5 mL/min. The gradient used was 0%–40% B in 40 min, with buffer A containing 20 mM Tris, 20 mM NaCl, pH 7.0 and buffer B the same as buffer A but with 500 mM NaCl, pH 7.0. For all chromatographic steps, UV detection was at 280 nm. Lastly, the homogeneous ACE-like peptidase (100 ng) was incubated with angiotensin I (30 µM) overnight, in 100 mM Tris, 50 mM NaCl, 10 µM ZnCl_2_ buffer, pH 7.0 and the hydrolysis was verified by HPLC, as described in Section 4.6.

### 4.10. Purified Protein Characterization

#### 4.10.1. SDS-PAGE—In-Gel Digestion and Mass Spectrometry

The active fractions of each purification step were analyzed by 13% polyacrylamide gel electrophoresis (SDS-PAGE), as previously described [55]. Samples were solubilized in non-reducing sample buffers, and protein profiles were visualized by silver stain.

The 70-kDa protein band corresponding to ACE-like peptidase, obtained after the last purification step, was subjected to an in-gel digestion with trypsin (Sigma-Aldrich, St. Louis, MO, USA) [56]. The mixture was then desalted, concentrated, and resuspended in 0.1% formic acid. Mass spectrometric analysis was performed by online liquid chromatography in an Easy-nLC Proxeon nanoHPLC system coupled with an LTQ-Orbitrap Velos (Thermo Fisher Scientific, Bremen, Germany) through a nanoelectrospray ion source. Raw data files were analyzed on PEAKS Studio (version 8.0, Bioinformatics Solution, Waterloo, ON, Canada) [53,54] against the library constructed with sequences deposited on UNIPROT with restriction to *Tityus* genus with addition of the ACE-like peptidase transcripts sequences from genus *Tityus* [17,30] (GenBank JAG85170.1, Tobs01141, TserSP00939), with a total of 383 sequences. A decoy database was also searched to calculate the false discovery rate (FDR) using the decoy-fusion method [53,54]. The search parameters were: trypsin cleavage specificity (max 1 missed cleavages); precursor mass tolerance set to 0.5 Da; and a fragment ion mass tolerance of 0.1 Da. Regarding Post Translational Modifications (PTM), we set carbamidomethylation as fixed modification and oxidized methionine, deamidation of asparagine and glutamine (NQ), and acetylation of *N*-term as the variable modifications. The peptide sequences that resulted from MS/MS were analyzed in Peaks DB and the matched peptides were filtered by FDR ≤ 1%, and protein confidence score −10lgP ≥ 62.

#### 4.10.2. Bioinformatic Analysis

The deduced sequences of ACE-like peptidase transcripts from the gland of *T. serrulatus* (GenBank TserSP00939), *T. bahiensis (*JAG85170.1) [17], and *T. obscurus* (GenBank Tobs01141) were aligned with human testicular ACE sequence (SwissProt) using MEGA 6 software (Arizona State University, Phoenix, AZ, USA, 2014). Identities and similarities were calculated using the SIAS server, aligned by “Length of Multiple Sequence Alignment” using the BLOSUM62 matrix.

## Figures and Tables

**Table 1:** Hydrolysis of biologically active peptides by *Tityus serrulatus* venom and released fragments.

Peptide	Fragment Sequence	Fragment Identification	MW	Venom Specific Activity (µM/µg/min)
Angiotensin I	DRVY	Ang_(1–4)_	551.2	0.050
	IHPFHL	Ang_(5–10)_	762.4	
	HPFHL	Ang_(6–10)_	649.3	
	DRVYIHP	Ang_(1–7)_	898.4	
	DRVYIHPF	Ang II	1045.4	
	RVYIHPF	Ang III	930.5	
	FHL	Ang_(8–10)_	415.2	
Bradykinin	RPPGF	BK_(1–5)_	572.3	0.045
	RPPGFSP	BK_(1–7)_	756.3	
	PGFSPFR	BK_(3–9)_	806.4	
Hemopressin	PVNFKFL	Hemo_(1–7)_	863.4	0.400
	PVNFKF	Hemo_(1–6)_	750.4	
	PVNFK	Hemo_(1–5)_	603.3	
	KFLSH	Hemo_(5–9)_	630.35	
	FLSH	Hemo_(6–9)_	502.25	

Assays were carried out in 100 mM Tris buffer containing 50 mM NaCl, 10 µM ZnCl_2_, pH 7.0, at 37 °C, in a final volume of 100 µL. The results are shown as the mean of three independent experiments. The SD of kinetic was never greater than 5% of the value obtained.

**Table 2:** Inhibition of *Ts*v activity on bioactive peptides by captopril and EDTA.

Peptide	Inhibitory Activity (%)		
	EDTA	Captopril 100 nM	Captopril 1 µM
Angiotensin-I	100	8.2	48.6
Bradykinin	100	36.3	60.0
Dynorphin A	100	0.0	1.8
Hemopressin	100	37.8	8.9

Experiments were made using 100 mM Tris, 50 mM NaCl, 10 µM ZnCl_2_ buffer, pH 7.0 at 37 °C with *Ts*v (1 µg), at 37 °C, using 30 µM of each biologically active peptide substrate. The incubations varied according to the particularity of each substrate, as described in Section 4.6. The results shown are the mean of three independent experiments. The SD of kinetic was never greater than 5% of the value obtained.

**Table 3:** Identity (white) and similarity (grey) between ACE-like from the venom of Brazilian *Tityus* sp. scorpions and *Homo sapiens* testicular ACE (AAA60611.1).

Proteins	TesticularACE *Homo sapiens* (AAA60611.1)	*T. serrulatus (*TserSP00939)	*T. bahiensis* (JAG85170)	*T. obscurus* (Tobs01141)
testicularACE *Homo sapiens* (AAA60611.1)		39.18%	23.12%	39.18%
*T. serrulatus (*TserSP00939 )	46.39%		67.21%	90.34%
*T. bahiensis* (JAG85170)	28.16%	67.34%		61.22%
*T obscurus* (Tobs01141)	46.53%	92.51%	62.31%	

## Supplementary Materials

The following are available online at www.mdpi.com/2072-6651/8/12/348/s1, Table S1: Summary of the purification protocol of Angiotensin-converting enzyme-like from *Tityus serrulatus* venom; Figure S1: Sequence alignment of testicular ACE from *Homo sapiens* (AAA60611.1) with venom ACE-like from the scorpions *Tityus serrulatus* (TserSP00939), *T. bahiensis* (JAG85170), and *Tityus obscurus* (Tobs01141). The metallopeptidase motif HEXXH is marked in black, peptides found by mass spectrometry are underlined, and conserved regions are highlighted in gray.
