# Supplementary material for: Insights into the Hypertensive Effects of Tityus serrulatus Scorpion Venom: Purification of an Angiotensin-Converting Enzyme-Like Peptidase
Source: Toxins (Basel). 2016 Nov 24;8(12):348. doi: 10.3390/toxins8120348 (PMC5198543; doi:10.3390/toxins8120348)
Supplement: Supplementary file 1 [file toxins-08-00348-s001.pdf]

# Supplementary Materials: Insights into Hypertensive Effects of the *Tityus serrulatus* Scorpion Venom: Purification of an Angiotensin-Converting Enzyme-Like Peptidase

**Daniela Cajado-Carvalho, Alexandre Kazuo Kuniyoshi, Bruno Duzzi, Leo Kei Iwai, Úrsula Castro de Oliveira, Inácio de Loiola Meirelles Junqueira de Azevedo, Roberto Tadashi Kodama and Fernanda Vieira Portaro**

**Table S1.** Summary of the purification protocol of Angiotensin-converting enzyme-like from *Tityus serrulatus* venom.

| Steps | Fraction | Volume (μL) | Protein (μg) | Activity (UF/min) | Total Activity (units* μL) | Specific Activity (units/μg) | Purification Factor | Yield (%) |
|---|---|---|---|---|---|---|---|---|
| Crude venom | Whole venom | 2000 | 25,000 | 1801 | 3,602,000 | 144.1 | 1.00 | 100 |
| DEAE | F1 | 500 | 778 | 1800 | 900,000 | 1156.8 | 8.03 | 100 |
| Gel Filtration | F1-2 | 500 | 61 | 161 | 80,500 | 1319.7 | 9.16 | 9 |
| PA-CM | F1-2.7 | 1500 | 10 | 22 | 33,000 | 3300.0 | 22.90 | 1 |

* versus

```
Homo sapiens (AAA60611.1)       EYDRTSQVVWNEYAEANWNYNTNITTETSKILLQKNMQIANHTLKYGTQARKFDVNQLQNTTIKRIIKKVQDLERAALPA [160]
Tityus serrulatus (TserSP00939) HANEVNEKQCNLFQTAYWNYDTNITIFNKNKMIQQQMIASNVSKKIWGVLTKFPWKKFSDPQLRRQFYQLSFLGDSALSD [160]
Tityus bahiensis (JAG85170)     HANEVNEKQCNLFQTASWNYDTDITILNKNKMIQQQMIASNVSKKIWGVLTKFPWKKFSDPQLRRQFYQLSFLGDSALSD [160]
Tityus obscurus (Tobs01141)     HVNEVNEKQCNLFQRASWNYDTNITIPNKNKMIEQQMIASNISKEMWKVLTKFQWKKFFDPQLRRQFYHLSFLGDSALPS [160]

Homo sapiens (AAA60611.1)       QELEEYNKILLDMETTYSVATVCHP---NGSCLQLEPDLTNVMATSRKYEDLLWAWEGWRDKAGRAILQFYPKYVELINQ [240]
Tityus serrulatus (TserSP00939) DKLRKKSSLEADMTKIYSTTTICDFTNKNKCNLSLDPDLSNILANSNNYYELLYVWKEWRNKVGRKIKPLYWEFVHLKNE [240]
Tityus bahiensis (JAG85170)     DKLRKKSSLEADMTKIYSTTTICDFTNKNKCNLSLDPDLSNILANSNNYYELLYVWKEWRNKVGRKIKPLYWEFVHLKNE [240]
Tityus obscurus (Tobs01141)     DKIRKKTSLEADMTNIYSTTTICDFKNKKKCNLSLEPDLSSIMANSNDYYELLHVWKEWRNKVGRKIRPLYWEFVDLKNE [240]

Homo sapiens (AAA60611.1)       AARLNGYVDAGDSWRSMYETPSLEQDLERLFQELQPLYLNLHAYVRRALHRHYGAQHINLEGPIPAHLLGNMWAQTWSNI [320]
Tityus serrulatus (TserSP00939) AARLNGFKNAGEFQREKYESPTLIQDLEDLWQQIRPLYQQLHAYVRRRLIEKYGNDKISADGPIPAHLLGNMWSQEWQNI [320]
Tityus bahiensis (JAG85170)     AARLNGFKNAGEFQREKYESPTLIQDLEDLWQQIRPLYQQLHAYVRRRLIEKYGNDKISAHGPIPAHLLGNMWSQEWQNI [320]
Tityus obscurus (Tobs01141)     AARLNGFNDAGEFQREKYESPTLIQDLEKLWHQIRPLYQQLHAYVRRKLIEMYGSDKIVKDGPIPAHLLGNMWSQEWQNI [320]

Homo sapiens (AAA60611.1)       YDLVVPFPSAPSMDTTEAMLKQGWTPRRMFKEADDFFTSLGLLPVPPEFWNKSMLEKPTDGREVVCHASAWDFYNGKDFR [400]
Tityus serrulatus (TserSP00939) INITIPYRNKPSLDVTPQMKAKGMKPVQIAKLAERFFVSLGLKPMTKEFWSNSLLEKPKD-RKVVCHASAWDLCNKRDFR [400]
Tityus bahiensis (JAG85170)     INITIPYRNKPSLDVTPQMKAKGMKPVQIAKLAEQFFVSLGLKPMTKEFWSNSLLEKPKD-RKVVCHASAWDLCNKRDFR [400]
Tityus obscurus (Tobs01141)     INITMPYRNKPSLDVTPQMKAKGMTPVQIAKLAEQFFVSLGLQPMTKEFWSNSLFEKPKD-RKVVCHASAWDLCDKRDFR [400]

Homo sapiens (AAA60611.1)       IKQCTTVNLEDLVVAHHEMGHIQYFMQYKDLPVALREGANPGFHEAIGDVLALSVSTPKHLHSLNLLSSEGGSDEHDINF [480]
Tityus serrulatus (TserSP00939) IKMCMETTMDFLITTHHEMGHVQYYMQYANQPHVFRKGANPGFHEAIGDVMSLSVYTPNHLQSIGLMNYTVSDREGDINF [480]
Tityus bahiensis (JAG85170)     IKMCMETTMDFLITTHHEMGHVQYYMQYADQPHVFRKGANPGKF*----------------------------------- [480]
Tityus obscurus (Tobs01141)     IKMCMECTMDFLITTHHEMGHVQYYMQYANQPHVFRKGANPGFHEAIGDVMSLSVYTPNHLQSIGLLNDIVSDREADINF [480]

Homo sapiens (AAA60611.1)       LMKMALDKIAFIPFSYLVDQWRWRVFDGSITKENYNQEWWSLRLKYQGLCPPVPRTQGDFDPGAKFHIPSSVPYIRYFVS [560]
Tityus serrulatus (TserSP00939) LLQMALKKIAFLPYGYILDKWRWGVYSGEVELDEWNTKWWEMRFKYQGVCPPVRRTEIDFDPGAKFHVPADTPYIRYFIS [560]
Tityus bahiensis (JAG85170)     -------------------------------------------------------------------------------- [560]
Tityus obscurus (Tobs01141)     LLQMALKKIAFLPFGYILDKWRWEVYSGEIEFEEWNRRWWEMRFKYQGVCPPIRRTEIDFDPGAKFHVPADTPYIRYFIS [560]

Homo sapiens (AAA60611.1)       FIIQFQFHEALCQAAGHTGPLHKCDIYQSKEAGQRLATAMKLGFSRPWPEAMQLITGQPNMSASAMLSYFKPLLDWLRTE [640]
Tityus serrulatus (TserSP00939) FIIQFQFHEALCRVTGFQGPLHKCDIYNSKEAGKLISEMMQMGSSRPWPEAFEKITGTRKMNANSLLEYFKPLYEWLKEA [640]
Tityus bahiensis (JAG85170)     -------------------------------------------------------------------------------- [640]
Tityus obscurus (Tobs01141)     FIIQFQFHEALCRVSGFQGPLHKCDIYNSKEAGKLISGMMQMGSSRPWPEAFEKITGSRKMNADSLLEYFKPLYEWLKEA [640]

Homo sapiens (AAA60611.1)       NELHGEKLGWPQYNWTPNSARSEGPLPDSGRVSFLGLDLDAQQARVGQWLLLFLGIALLVATLGLSQRLFSIRHRSLHRH [720]
Tityus serrulatus (TserSP00939) NK--NETIGWNTIDPTVCP------------------------------------------------------------- [720]
Tityus bahiensis (JAG85170)     -------------------------------------------------------------------------------- [720]
Tityus obscurus (Tobs01141)     NK--NEVIGWETFDPTVCP------------------------------------------------------------- [720]

Homo sapiens (AAA60611.1)       SHGPQFGSEVELRHS [735]
Tityus serrulatus (TserSP00939) --------------- [735]
Tityus bahiensis (JAG85170)     --------------- [735]
Tityus obscurus (Tobs01141)     --------------- [735]
```

**Figure S1.** Sequence alignment of testicular ACE from *Homo sapiens* (AAA60611.1) with venom ACEs from the scorpions *Tityus serrulatus* (TserSP00939), *T. bahiensis* (JAG85170) and *Tityus obscurus* (Tobs01141). The metallopeptidase motif HEXXH is marked in black, peptides found by mass spectrometry are underlined and conserved regions highlighted in grey.
